# Modeling Early Radiation DNA Damage Occurring During ^177^Lu-DOTATATE Radionuclide Therapy

**DOI:** 10.2967/jnumed.121.262610

**Published:** 2022-05

**Authors:** Giulia Tamborino, Yann Perrot, Marijke De Saint-Hubert, Lara Struelens, Julie Nonnekens, Marion De Jong, Mark W. Konijnenberg, Carmen Villagrasa

**Affiliations:** 1 Research in Dosimetric Applications, Belgian Nuclear Research Centre, Mol, Belgium;; 2 Department of Radiology and Nuclear Medicine, Erasmus MC Cancer Institute, Erasmus University Medical Center, Rotterdam, The Netherlands;; 3 IRSN, Institut de Radioprotection et de Sûreté Nucléaire, Fontenay aux Roses, France; and; 4 Department of Molecular Genetics, Oncode Institute, Erasmus MC Cancer Institute, Erasmus University Medical Center, Rotterdam, The Netherlands

**Keywords:** DNA double-strand break simulation, targeted radionuclide therapy, ^177^Lu-DOTATATE, dose–effect relationship, Geant4-DNA

## Abstract

The aim of this study was to build a simulation framework to evaluate the number of DNA double-strand breaks (DSBs) induced by in vitro targeted radionuclide therapy (TRT). This work represents the first step toward exploring underlying biologic mechanisms and the influence of physical and chemical parameters to enable a better response prediction in patients. We used this tool to characterize early DSB induction by ^177^Lu-DOTATATE, a commonly used TRT for neuroendocrine tumors. **Methods:** A multiscale approach was implemented to simulate the number of DSBs produced over 4 h by the cumulated decays of ^177^Lu distributed according to the somatostatin receptor binding. The approach involves 2 sequential simulations performed with Geant4/Geant4-DNA. The radioactive source is sampled according to uptake experiments on the distribution of activities within the medium and the planar cellular cluster, assuming instant and permanent internalization. A phase space is scored around the nucleus of the central cell. Then, the phase space is used to generate particles entering the nucleus containing a multiscale description of the DNA in order to score the number of DSBs per particle source. The final DSB computations are compared with experimental data, measured by immunofluorescent detection of p53-binding protein 1 foci. **Results:** The probability of electrons reaching the nucleus was significantly influenced by the shape of the cell compartment, causing a large variance in the induction pattern of DSBs. A significant difference was found in the DSBs induced by activity distributions in cell and medium, as is explained by the specific energy ($\bar{z}$) distributions. The average number of simulated DSBs was 14 DSBs per cell (range, 7–24 DSBs per cell), compared with 13 DSBs per cell (range, 2–30 DSBs per cell) experimentally determined. We found a linear correlation between the mean absorbed dose to the nucleus and the number of DSBs per cell: 0.014 DSBs per cell mGy^−1^ for internalization in the Golgi apparatus and 0.017 DSBs per cell mGy^−1^ for internalization in the cytoplasm. **Conclusion:** This simulation tool can lead to a more reliable absorbed-dose–to–DNA correlation and help in prediction of biologic response.

## Visual Abstract

**Figure:**
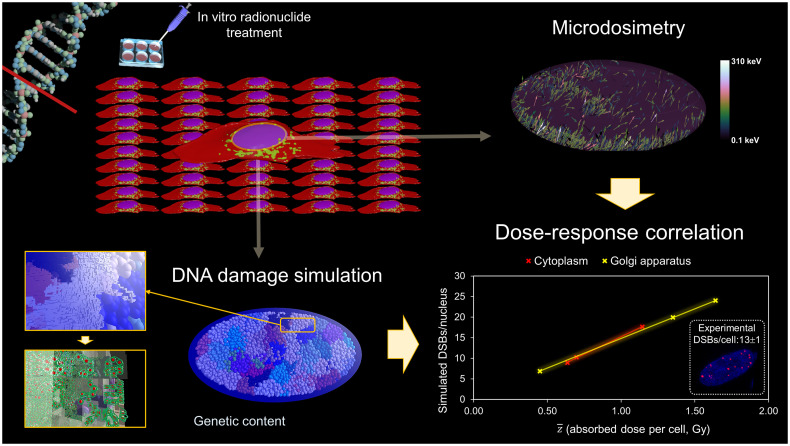

The most common way of exposing cancer patients to radiation is through external-beam radiotherapy (EBRT). The success and effectiveness of EBRT can, at least partially, be attributed to knowledge of its radiobiologic principles and their integration into dose–response modeling (1).

An alternative form of anticancer therapy is targeted radionuclide therapy (TRT). TRT is based on injection of a radiolabeled molecule that has the advantage of targeting specific cancer cells, enabling delivery of a cytotoxic absorbed dose to eradicate both a primary tumor site and metastases (2).

In striking contrast to EBRT, TRT is marked by a scarcity of radiobiologic investigations and dose–response modeling. The physical characteristics of TRT—that is, heterogeneous radiation caused by variable uptake at cellular and subcellular levels, protracted exposure causing overlapped biologic mechanisms such as DNA damage formation and repair, and low dose-rate—differ significantly from those of EBRT. Hence, TRT-specific radiobiologic knowledge and biophysical modeling need to be developed (3).

The initial step into understanding the cell’s radiobiologic response is represented by calculation of the energy deposition on a subcellular scale and, in particular, in the cell nucleus, where radioinduced DNA damage can be considered a key biologic output for predicting cellular fate (4). Ultimately, a mechanistically informed model, including the cell’s response dependence on phenotype, cell cycle, microenvironment, type of radiation, and delivery method, would elucidate the underlying biologic mechanisms and hence allow prediction of the radiosensitivity of individual tissues under a particular irradiation condition (5).

DNA is recognized as a key target, and currently, simulations of in vitro DNA damage in the context of TRT have been focused primarily on low-energy electrons, namely Auger electrons (e.g., ^125^I-iodo-2′-deoxyuridine,^111^In-DTPA-d-Phe^1^-octreotide, and ^64^CuCl_2_), because of their significant decrease in energy density as a function of distance in nanometers (6).

Various models of DNA target, ranging from DNA linear fragments represented by structured cylinders (7) to either simplified (8) or complex atomic representations (9,10), have been applied for this purpose using various Monte Carlo codes. On the other hand, a combination of precalculated cluster DNA damage yields by Monte Carlo damage simulation code (11) and local dose distributions within a local effect model has been used as alternative fast approach (12).

For Auger emitters internalized in the nucleus, the choice of DNA model and the placement of the radionuclide with respect to the DNA structure are the main parameters influencing the resulting double-strand break (DSB) computation (10) because of their nanometer range. As a consequence, cell morphology and cell population are not modeled in this scenario. On the contrary, longer-range radionuclides, such as ^177^Lu, require a detailed cell morphology and population modeling to account for both self- and cross-irradiation in a planar cell colony (13). Furthermore, once the irradiation field has been characterized, an event-by-event description of the radiation track structure at the nanometer level within the nucleus, combined with a simulation including a description of the target at the relevant scale (e.g., atom, molecule), needs to be adopted in order to yield conclusions on the biophysical mechanisms involved. In this respect, faster Monte Carlo approaches for DSB simulation, intrinsically relying on uniform external irradiation parameters, would not provide a deeper understanding of the mechanisms involved and, as such, would not help to contribute to the final goal of developing methods to select the best approach to individualized treatment optimization. In a similar way, nanodosimetric simulations calculating the ionization cluster size distributions in water cylinders corresponding to DNA segments (14) rely on adjustable parameters—inferred from EBRT exposure—to account for the missing geometric DNA details and, hence, would not completely serve this purpose.

A successful example of TRT, leading to markedly prolonged survival and an increased quality of life in comparison to nonradioactive targeted therapy (15*,*^16^), is ^177^Lu-DOTATATE. ^177^Lu-DOTATATE treatment targets tumor cells overexpressing the somatostatin receptor type 2 (SSTR_2_) and is authorized in Europe and the United States as Lutathera (Advanced Accelerator Applications) for therapy of metastasized neuroendocrine tumors (17).

This work proposed a simulation framework evaluating the number of DNA DSBs occurring during in vitro ^177^Lu-DOTATATE experiments with planar colonies, thereby accounting for detailed cellular morphologies and source localizations. We analyzed the impact of different modeling assumptions and compared them with experimental data. This study represents a first step toward a better understanding of the underlying biologic mechanisms of ^177^Lu-DOTATATE exposure by providing a detailed description of early DSB distribution.

## MATERIALS AND METHODS

A 2-step simulation process was adopted aiming to model, first, the internal irradiation setup, characterizing particles entering the nucleus belonging to a planar colony, and, second, the DNA damage induced in that specific nuclear shape. The uptake assay, immunofluorescent staining, and imaging of the cellular morphologies were previously established (13*,*^18^), and details are described in the supplemental materials (available at http://jnm.snmjournals.org).

### Modeling the Internal Irradiation Setup

**FIGURE 1.**
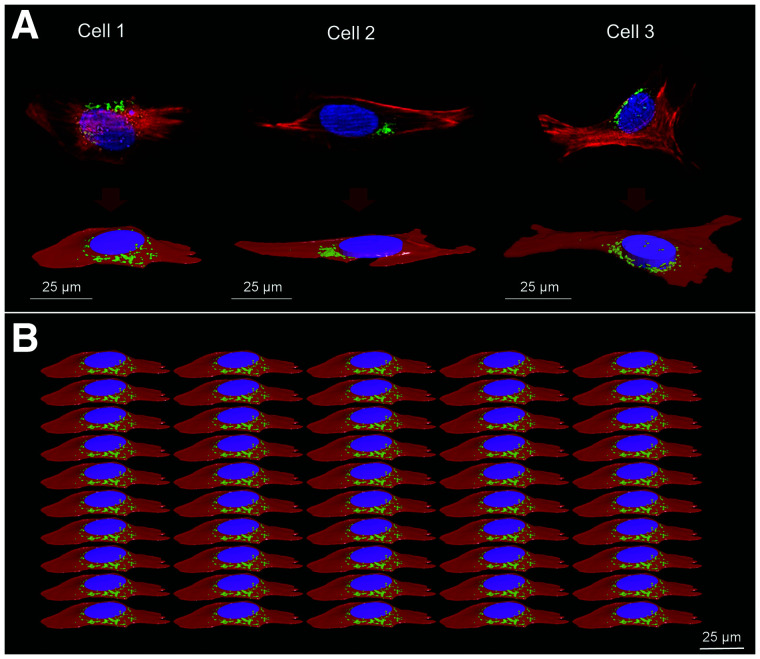
Cellular morphologies. (A) 4Pi confocal microscope images with corresponding polygonal mesh structures. (B) Example of cell population representing modeled planar cellular cluster in Geant 4 (perspective view) where all cells are identical. Nucleus, G, and Cy are represented in blue/purple, green, and red, respectively. Cell population models reproduce confluence level of 50% ± 5%, estimated from radiobiologic observations. Geometric characteristics of the 3 cells are reported in Table 1.

Cellular polygonal mesh models from representative 4Pi confocal microscopic images of human osteosarcoma cells (U2OS-SSTR_2_) were used to model 3 cellular morphologies in Geometry Description Markup Language format. Each cellular shape consists of the cellular membrane (CM), cytoplasm (Cy), Golgi apparatus (G), and nucleus. The nucleus was simplified by either an ellipsoid or an elliptic cylinder, preserving its original volume and proximity to the other cellular compartments (Fig. 1A). The geometric characteristics of the 3 cells are summarized in Table 1.

**TABLE 1.** Geometric Characteristics of the 3 Cell Morphologies

	Volume (μm^3^)		
Parameter	Cell 1	Cell 2	Cell 3
Cy	3,465.64	1,876.58	4,228.08
G	68.46	24.34	63.18
Nucleus	811.79	714.71	1,105.84
Size* (μm)			
Cy	Bounding box: *x* = 72.24, *y* = 31.78, *z* = 5.99	Bounding box: *x* = 99.21, *y* = 30.86, *z* = 3.52	Bounding box: *x* = 88.70, *y* = 64.28, *z* = 6.29
Nucleus	Ellipsoid: a = 12, b = 8.5, c = 1.9	Elliptic cylinder: a = 13, b = 7, c = 1.25	Elliptic cylinder: a = 8, b = 11, c = 2

* Reported in half-dimensions for nucleus.

CM thickness = 0.0075 μm (42*,*^43^).

Simulations were performed on Geant4.10.06 (19–21). A parameterization process replicating each cellular shape, and its subcompartments, within an array was used to create 3 planar populations of 50 adjacent cells of the same shape (Fig. 1B). The number of cells was chosen to allow a cell layer dimension greater than the average range of ^177^Lu β particles (continuous slowing down approximation range at average energy and maximum energy = 270 μm and 1.76 mm, respectively). Indeed, the ^177^Lu cross dose (i.e., the absorbed dose delivered by surrounding cells to a target cell) decreases exponentially with distance, and hence, increasing the planar cellular cluster size with additional cell layers after a given value (on average, 3–4 cell layers) would not significantly contribute to the total absorbed dose received by the nucleus of the central cell (13). The computational memory consumption was drastically reduced by the Geant4 parameterization process, since the tessellated geometries (i.e., polygonal mesh) used to model each cellular morphology were stored only once in the memory. The cells were attached to the bottom of a water cylinder.

The decay spectrum of ^177^Lu is reported in Supplemental Table 1 for reference. In this study, the full continuous radar (β) and discrete internal conversion (IC) electron (ICRP107) spectra were simulated, whereas photon and Auger electron emissions were neglected. Photon emissions are considered negligible for cellular dosimetric purposes (22), and Auger electrons are unlikely to reach the nucleus from Cy or G and CM. Each of these source components was sampled separately in order to distinguish the contribution of β and IC electrons coming from the same nuclear transition.

**FIGURE 2.**
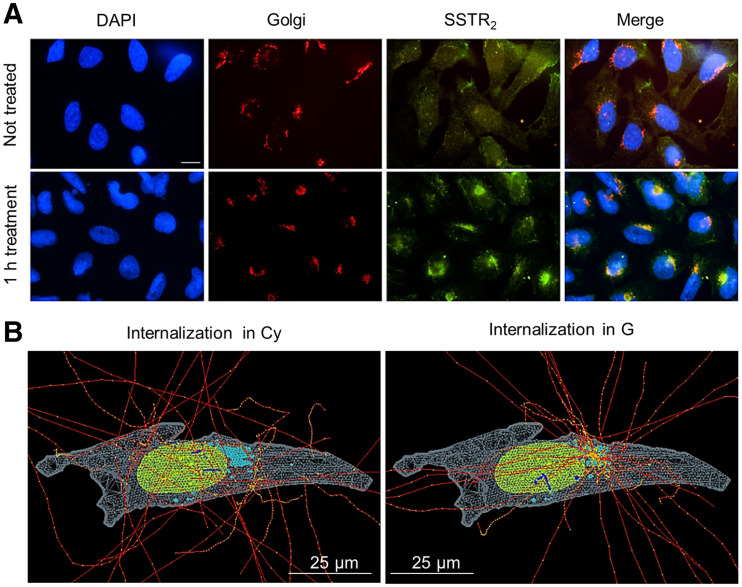
Immunofluorescent staining of U2OS-SSTR_2_ cells and corresponding simulation hypotheses. (A) From left to right, images report nucleus, G, and SSTR_2_ stainings for untreated cells (top) and cells incubated with DOTATATE (bottom). Merged image at end highlights colocalization of SSTR_2_ with G after 1 h of incubation with DOTATATE. Scale bar = 5 µm. (B) Example of internalized source simulation for cell morphology 2. Nucleus, G, and Cy are reported in green, light blue, and light gray, respectively. Electron tracks are drawn in red, with yellow energy deposition points, which become blue when traversing nucleus.

The radioactive source (^177^Lu-DOTATATE) was assumed to be instantly and permanently incorporated within the cell (internalized), whereas a smaller portion remained membrane-bound on the basis of the uptake measurements. The probability of emission within the cell (73%) or the membrane (27%) was sampled according to previous uptake experiments with 2.5 MBq/mL (13), and hence, following the average cell population behavior. Two internalization hypotheses (i.e., G or Cy) were investigated (Fig. 2), and because of the impossibility of distinguishing an intraorganelle variation in the activity distribution, the activity was sampled uniformly in each cell compartment (G, Cy, and CM). The radioactive source was sampled in all cells simultaneously.

The unspecific contribution of the medium to DSB induction was investigated in a separate simulation for 1 nuclear geometry (cell 1), given that the absorbed dose from medium to nucleus is not significantly influenced by the nuclear volume. Here, the source was uniformly distributed in a cylinder with a size corresponding to the maximum range of ^177^Lu-β particles (diameter and height, 1.76 mm).

The Livermore low-energy physics models were adopted in Geant4 to track electrons down to an energy of 100 eV, and the default production threshold of secondary electrons was set to 0.2 μm (adapted to cell nuclear volumes), which corresponds to 1.75 keV in liquid water. Atomic deexcitation processes, such as Auger cascades and fluorescence, were included in the simulations. The chemical composition of CM, Cy, G, and the nucleus was the same as water (ρ = 1 g/cm^3^) (National Institute of Standards and Technology database). The position, direction, energy, compartment of emission, and event identifier, which identifies particles derived from the same primary, were recorded for each particle entering the nucleus of the central cell, assumed as representative for the cell population. The number of particles run per simulation ensured a phase space file larger than 1 million particles.

### DNA Damage Simulation

DSB yield calculations were performed with a computational chain (23) using the Geant4-DNA (24–27) extension of the Geant4 toolkit (version 10.1). In this case, all electron interactions are simulated in a discrete manner (i.e., step-by step) down to the electron thermalization, making possible the track structure simulation required at the nanometer scale; as such, they simulate explicitly all interactions and do not use any production cut. The simulation chain includes not only these physical interactions but also the physicochemical and chemical stages within a representative cell nucleus with DNA structure (Supplemental Fig. 1). Therefore, simulations were performed to compute DNA strand breaks (i.e., direct damage of the DNA backbone and indirect damage of the DNA backbone-sugar leading to strand breaks). DSBs were scored from the simulated strand breaks as defined previously (13), that is, at least 2 strand breaks located in opposite strands and separated by less than 10 base pairs. The genomic content of cell nuclei composed of chromatin fibers in the G0/G1 phase of the cell cycle was generated with the DNAFabric software (28*,*^29^). The simulation chain coupled to these geometries allowed calculation of DSBs per source particle (SP) reaching the nucleus, as recorded in the phase space file. Source particles characterized by the same event identifier, whose tracks are related to the same primary, were simulated together until a relative SD of 5% on the average DSBs per SP was reached. The DSB yields are reported in terms of DSBs per SP and per gigabase pair (Gbp) ($N_{\text{DSBs/}(\text{SP}\;\text{Gbp})}$), and for the calculations of total number of DSBs, all nuclei are assumed to have 6 Gbp ($N_{\text{DSBs/SP}}$) (where *N* [within the DSB formulas] or *n* [when neither uppercase nor lowercase letter is subscripted] is *number* and where *N* [when subscripted and following the arrow] indicates *nucleus*).

### DSB Calculations and Measurement

The output of the DSB simulations ($N_{\text{DSBs/SP}}$) is converted to the number of DSBs corresponding to an added activity of 2.5 MBq/mL, as follows:

$$N_{\text{DSBs}\;}={\left(\left(n_{M}\;p_{M\to N}+n_{C}\;p_{C\to N}\right)\;\times \;N_{\text{DSBs/SP}}\right)}_{\text{β}}+0.15\;{\left(\left(n_{M}\;p_{M\to N}+n_{C}\;p_{C\to N}\right)\;\times \;N_{\text{DSBs/SP}}\right)}_{\text{IC-electrons}},$$

where $n_{M}$ and $n_{C}$ are the number of decays cumulated in a time interval within the medium and the cells (membrane-bound and internalized), respectively; and $p_{M\to N}$ and $p_{C\to N}$ are the probabilities that emissions from medium or cells will reach the nucleus of the central cell. The factors related to the cell contribution ($n_{C}\;p_{C\to N}$) comprise either G or Cy irradiation. The total $N_{\text{DSBs}\;}$ are then calculated accounting for the contribution of β and IC electrons, weighting on the corresponding probabilities of emission from ^177^Lu (i.e., 1 and 0.15 per decay, respectively).

The simulated results are compared with the experimental number of DSBs per cell measured by p53-binding protein 1 (53BP1) foci formation as previously reported (18). Briefly, Z-stack imaging was performed using a TCS SP5 confocal microscope (Leica), and foci were counted from at least 50 cells of 2 independent experiments using Image J software (30) (settings: median blur, 1.0; maximum projection and find maxima; noise tolerance, 75 for cells and 100 for slices). The untreated average DSB level was subtracted from the measured data.

### Detailed Dosimetric Characterization of the Nucleus Irradiation

Source particles entering the nucleus of the central cell for each phase space file were compared in terms of energy and position/direction of entrance in the nucleus.

Furthermore, separate simulations scoring the specific energy within the nucleus were performed for each phase space file in order to justify the possible difference in DSB yields. Indeed, the determination of the energy distribution (and hence the macroscopic linear energy transfer) of electrons entering the nucleus alone is not sufficient to characterize the relationship of the electron tracks to biologic effectiveness. For this purpose, Geant4-DNA models and processes (physics list option 2) were used to enable track-structure (i.e., step-by-step) simulations of electrons in liquid water down to the millielectronvolt energy range. The energy deposited event by event within the nucleus was used to score the specific energy distribution.

### Statistical Analysis

The unpaired *t* test (2 group samples) and 1-way ANOVA (more than 3 group samples) were used to assess the significant difference (*P* < 0.05) between sets of data (DSB yields, probabilities to reach the nucleus, number of SPs traversing the nucleus) within the shape modeling and source localization comparison. A 2-way ANOVA was performed as well, to learn how cellular shapes and source localizations, in combination, affect these sets of data.

## RESULTS

### Cellular Shape and Internalization Compartment Influence Level of Nucleus Irradiation

**FIGURE 3.**
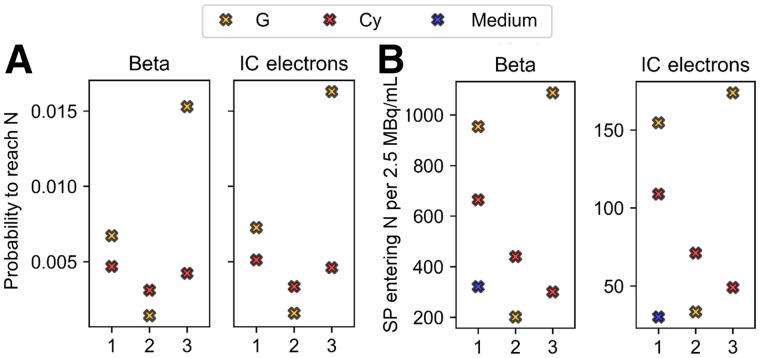
Comparison between probabilities (A) and number (B) of SPs entering nucleus for 3 cell models, as indicated by *x*-axis, and the 3 source localizations (Cy, G [including contribution of CM], and medium when comparable to cell sources), including planar cross-irradiation. Number of particles entering nucleus refers to 2.5 MBq/mL of added activity to which experimental data correspond. Medium contribution is assumed to be same for the 3 morphologies on basis of simulations for cell 1. Each graph is subdivided into 2 windows corresponding to the 2 emission types (β and IC), as indicated by titles. N = nucleus.

The probability of reaching the nucleus from the cell ($p_{C\to N}$) is 3–4 times higher than from the medium ($p_{M\to N}$) because of geometric factors. Moreover, $p_{C\to N}$ significantly depends on the proximity and distribution of the cell compartment with respect to the nucleus, as shown in Figure 3. The more the radioactive cell compartment closely surrounds the nucleus, the more $p_{C\to N}$ increases, as for G in cell 3.

To include the contribution of the medium in the previous comparison, Figure 3B reports the number of ^177^Lu disintegrations reaching the nucleus per particle type and cell corresponding to 2.5 MBq/mL of added activity. Once again, cell morphology and source location have a strong combined effect on the number of tracks reaching, and hence potentially damaging, the nucleus and its genetic content.

### DSB Induction Is Significantly Different When ^177^Lu Is Located Inside Cell or in Medium

**FIGURE 4.**
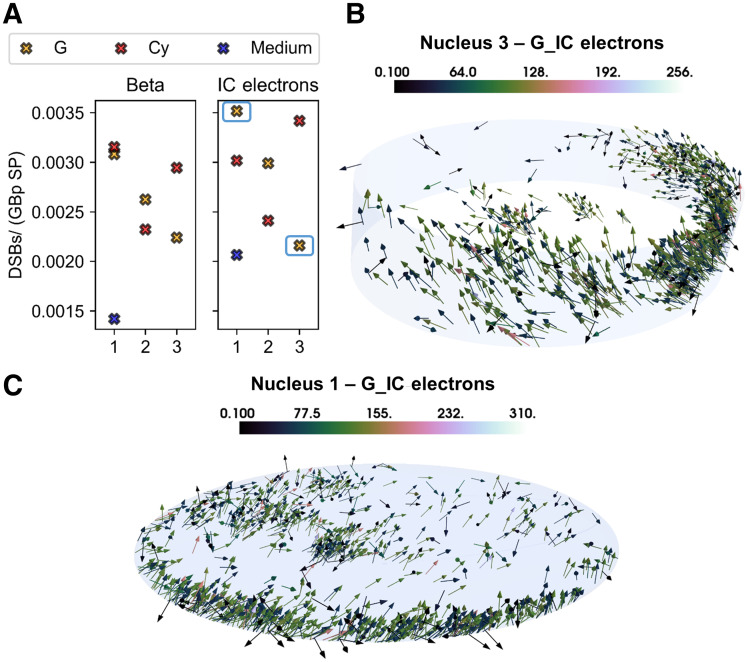
Simulation results and graphical explanation. (A) DSB-yield (DSBs/Gbp SP) comparison for the 3 cell morphologies (as indicated by *x*-axis), the 3 source localizations (Cy, G [including contribution of CM], and medium), and the 2 emission types. Medium contribution is assumed to be same for the 3 morphologies on basis of simulations for cell 1. (B) Total nucleus irradiation (i.e., self- and cross-irradiation) characterizing nucleus 3 when IC electrons are emitted from G. (C) Total nucleus irradiation (i.e., self- and cross-irradiation) characterizing nucleus 1 when IC electrons are emitted from G. Color bars indicate energy (keV) at entrance of nucleus.

The DSB yields normalized to the amount of genetic material (Gbp) and SPs reaching the nucleus ($N_{\text{DSBs/}(\text{SP}\;\text{Gbp})}$) differed significantly depending on the irradiation geometry (i.e., source and target shape and size in relation to particle track). The DSBs induced by the β particles in the medium are significantly lower than the ones induced by the 3 cell sources. Furthermore, even though the difference among the 3 cell morphologies is not significant, the localization (i.e., G or Cy) and the specific shape of the radioactive cell compartment cause a spread in the biologic damage, as shown in Figure 4A.

The variation in DSB yields among the analyzed cells is caused by the position and direction of particles entering the nucleus, which significantly depend on the cellular morphology. These characteristics affect the hit probability, which is the probability of having an energy deposition event potentially damage the DNA structure. Specifically, the broader angular distribution of the scattered β particles entering the nucleus from the medium increases the electrons that traverse it with a lower efficiency (greater polar angle). The same applies to the comparison between cell morphologies and cell compartments (Figs. 4B and 4C); in this case, the difference is predominantly less noticeable, given the overall similar source-to-nucleus proximity. Indeed, the proportion of events damaging the internalized source ranges from 0.91 to 0.93 for nucleus 1, from 0.45 to 0.62 for nucleus 2, and from 0.46 to 0.67 for nucleus 3, depending on internalization hypothesis and emission type. If the source is in the medium, the same range is reduced to 0.39–0.49. To understand these differences, we analyzed the distribution of energy deposition events in the nucleus by means of microdosimetric simulations.

When the DSB yields are divided by the mean absorbed dose delivered per particle source in each nucleus, the number of DSBs/(Gy Gbp SP) ranges between 2.3 and 3.0, depending on internalization hypothesis and particle type.

In terms of DSB complexity, that is, the number of close strand breaks that can be attributed to the same DSB, there is no significant difference among cell morphologies and type of particle emitted (β vs. IC electrons). The proportion of simple DSBs (i.e., DSBs made of 2 single-strand breaks) ranges between 79.7% and 92.2% with respect to the complex DSBs (i.e., DSBs made of 3 or more single-strand breaks, with at least 1 of them located in a strand opposite from the others), as expected for radiation with low linear-energy transfer.

### Specific Energy in Nucleus Explains DSB Yield Difference Between Cell and Medium Source

**FIGURE 5.**
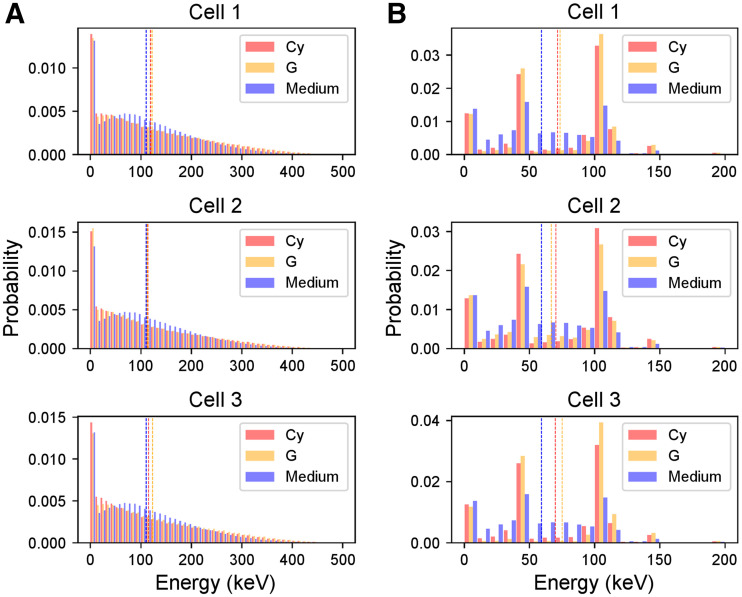
Energy spectra of electrons entering nucleus of the 3 cell morphologies. (A) Distributions corresponding to β particles. (B) Distributions corresponding to IC electrons. Each color corresponds to the 3 source localizations (Cy, G [including contribution of CM], and medium). Dotted lines indicate mean value of energy spectra. Spectrum of medium is assumed to be same as cell 1 for the 3 morphologies and is replicated in each graph for comparison with cell sources. Energy bin is 10 keV.

**FIGURE 6.**
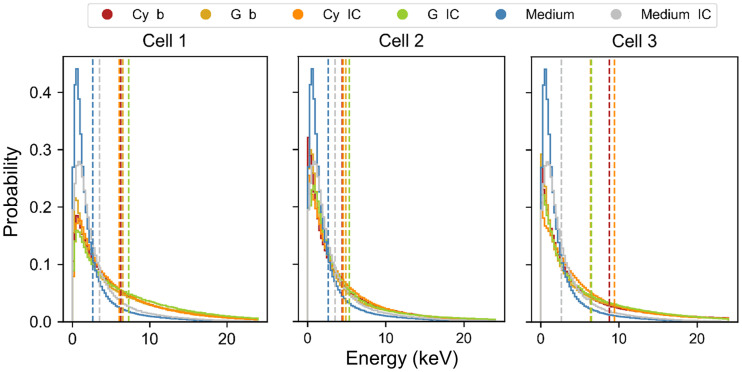
Probability density functions of energy deposited per particle in nucleus of the 3 cell morphologies. Each distribution corresponds to the 3 source localizations (Cy, G [including contribution of CM], and medium) and the 2 emission types (β and IC). Dotted lines indicate mean value of microscopic energy distributions, from which mean specific energy ($\bar{z}$) is evaluated (Table 2). Spectrum of medium is assumed to be same as cell 1 for the 3 morphologies and is replicated in each graph for comparison with cell sources.

The source localization does not significantly affect the energy distributions of particles entering the nucleus (Figs. 5A and 5B), explaining why the DSB range/Gy Gbp is similar for all cell morphologies. Indeed, the energy distributions of electrons coming from medium or cells, and hence their slowing down, are similar as well, between the β electrons and the IC electrons. However, the nucleus geometry affects the electron pathlength, causing significant differences in the energy deposition patterns within the nucleus itself (Fig. 6).

Specifically, the microdosimetric energy spectrum of particles coming from the medium is significantly shifted to lower energies with respect to all the sources (Fig. 6; Table 2), reflecting the DSB yield comparison. The difference among cell morphologies is also the result of these spectral differences, as the difference is evident when comparing the corresponding specific energy in Table 2 with the DSB yields in Figure 4.

**TABLE 2.** Mean Specific Energy per Particle Entering Nucleus of the 3 Cell Morphologies

	$\bar{z}$ (Gy)		
Parameter	Cell 1	Cell 2	Cell 3
Cy β	1.24	0.99	1.27
G β	1.29	1.10	0.92
Cy IC	1.20	1.02	1.36
G IC	1.44	1.20	0.94
Medium β	0.52		
Medium IC	0.69		

Medium values (β and IC electrons) are calculated for nucleus 1 and assumed same for the 3 morphologies.

### Simulated DSBs Match Experimental Data

**FIGURE 7.**
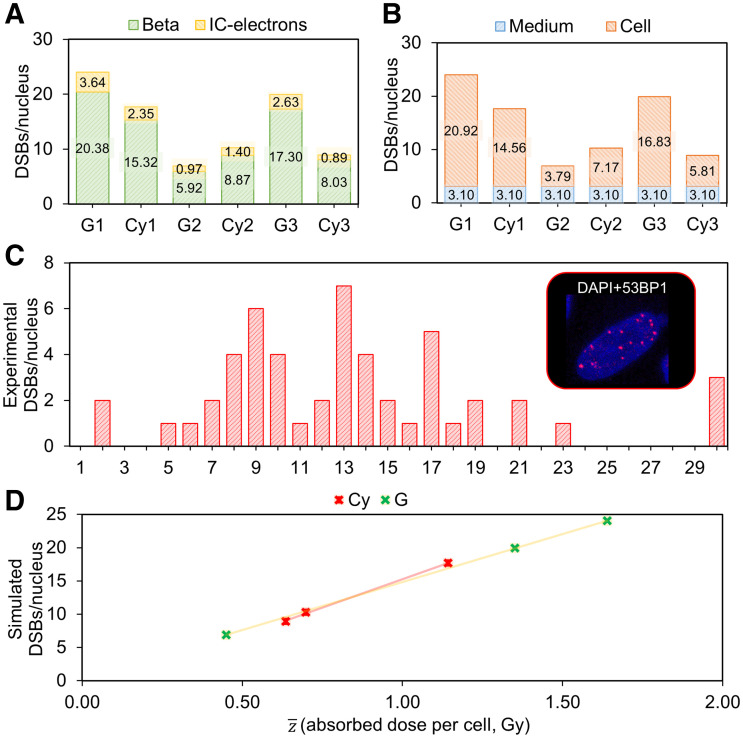
DSB simulations, comparison with experimental data, and correlation with absorbed dose to nucleus. (A) Simulated number of DSBs per nucleus corresponding to the 3 cell morphologies and internalization hypotheses (Cy vs. G, including CM), indicating contribution of each particle type (β and IC electrons). (B) Simulated DSBs per nucleus corresponding to the 3 cell morphologies and internalization hypotheses (Cy vs. G, including CM), indicating contribution of medium or cell source (internalized and membrane-bound). (C) Frequency histogram of experimental number of DSBs per nucleus induced by 4 h of administration of 2.5 MBq/mL activity of ^177^Lu-DOTATATE, measured by 53BP1. (D) Linear correlations between absorbed dose to nuclei and simulated number of DSBs when internalized source is located in Cy and in G. DAPI = 4′,6-diamidino-2-phenylindole.

The total number of simulated DSBs per cell for a 2.5 MBq/mL dose of ^177^Lu-DOTATATE ranges between 7 and 24 (Figs. 7A and 7B), compared with a range of 2–30 experimentally determined (Fig. 7C) (18). The mean DSBs per cell correspond to 14 and 13 for simulations and experiments, respectively.

As expected, the DSBs are induced mostly by β particles, whereas the IC-electron component results are significant only for specific cellular morphologies (Fig. 7A). The medium contribution is not assumed to vary depending on the nuclear geometry; hence, its relative impact depends strictly on the cell source contribution to each morphology (Fig. 7B).

Linear correlations (*R*^2^ = 1) with slopes of 0.014 and 0.017 DSBs per cell mGy^−1^ are found between the average specific energy and the simulated number of DSBs, when assuming the internalized source in G or Cy, respectively (Fig. 7D). Absorbed dose values corresponding to 2.5 MBq/mL are reported in Supplemental Table 2, and the absence of correlation when using average absorbed dose calculations is highlighted in Supplemental Figure 2.

## DISCUSSION

Modeling of DNA damage after TRT exposure can lead, through comparison with experimental data, to a better understanding of the underlying mechanisms of this treatment modality. Ultimately, it will allow evaluation of treatment efficacy, granting the flexibility of a simulation environment and, as such, new opportunities for the evaluation of novel radiopharmaceuticals. The first step toward this aim was made here, in which we accounted for detailed cellular morphologies and activity distributions to replicate a typical (^177^Lu-DOTATATE) planar in vitro TRT environment and tested the feasibility of performing DSB simulations through a simulation chain created for external radiation exposure.

The importance of an improved cellular morphology modeling has already been highlighted for macrodosimetric calculations (i.e., S values) involving a planar colony of cells exposed to ^177^Lu-DOTATATE (13); however, its impact on DSB yields had never, to our knowledge, been assessed before. Noticeably, detailed cellular morphology modeling and activity localization sampling were indispensable in correctly estimating the number of induced DSBs, since they significantly influence the probability that electrons will reach the nucleus and the distribution of track lengths within the nucleus itself. The volumetric and shape characterization of the nucleus is fundamental to correctly evaluate the energy deposition pattern as well.

Interestingly, the energy distributions of electrons entering the nucleus from the medium are not shifted to lower energies with respect to the cell source. For this reason, the difference in DSB yields induced by unbound (i.e., medium) and bound (i.e., cell) activity is not caused by different energy spectra of particles entering the nucleus. Indeed, in our simulations, the portion of electrons with energy below 10 keV, that is, the electrons with the highest relative biological effectiveness for DSB induction, were found to be very similar (within 1%) in all phase space files, indicating a possible similarity in the relative biological effectiveness for DSB induction (31). Instead, the energy deposition pattern within the nucleus reflected the difference in DSB yields, underlying the importance of both microdosimetric analysis and activity characterization on a cellular scale to predict biologic effects for radiation of low linear-energy transfer as well. Microdosimetry, in fact, accounts for the characteristics of the electron tracks (i.e., finite range and change of linear-energy transfer along the track, energy-loss straggling, δ-ray escape, and angular scattering) in order to correctly evaluate the concentration of energy transferred to the nucleus and hence the biologic effectiveness of the SPs.

Altogether, the need for detailed cellular morphology modeling, accurate sampling, and a microdosimetric framework able to explain biologic effects, as highlighted in this work, is in striking contrast to the current dosimetric approach of implementing simplifying cell models (i.e., concentric spheres) and a semianalytic radiation transport model adopting the continuous-slowing-down approximation (32). Indeed, not accounting for the typical complexity and heterogeneity at the cellular or multicellular levels and relying on averaged large-scale dosimetry might be the reason for missing dose–response correlations that could be translated on a clinical scale.

To our knowledge, this was the first study simulating DSB formation after ^177^Lu-DOTATATE exposure while including all stages of damage induction; hence, we compared our results with photons (producing similar secondary electron spectra) and electron beam irradiation data available in the literature. The number of DSB yields in this work (2.3–3.0 DSBs/Gbp^−1^ Gy^−1^ SP^−1^) was comparable to that in a study by Tang et al. (33), in which the simulated results ranged between 3.5 and 2.8 DSBs/(Gbp Gy) for 220-kVp and 4-MV x-ray irradiations, respectively. Similarly, Nikjoo et al. (34) estimated a DSB yield of 3.32 DSBs/(Gy Gbp) for 100-keV electrons, assuming 6 Gbp of genetic material and 3.9 10^−12^ Da/cell. In both cases, the portion of complex DSBs was similar to this study. In this work, the DSB complexity was independent from the source localization. Hence, repair mechanisms acting on DSBs caused by medium or cell source will most likely be the same.

The parameters implemented in the simulations to score DNA damage induction can strongly influence the final DSB yields. The good agreement reached with the aforementioned studies could be explained by the similar parameters set to score direct or indirect strand breaks. Indeed, increasing the chemical simulation end-time from 2.5 ns (as set in this work) to 10 ns would increase the number of DSBs by a factor of approximately 1.3 (33), and either decreasing the threshold for direct single-strand break induction from 17.5 eV to lower values or introducing a linear probability of between 5 and 37.5 eV would significantly affect the total number of DSBs (34*,*^35^). We did not study how these parameter variations would affect our calculations, given that our results were already comparable to the experimental data (18) and that the computational time required for these simulations is considerably long.

Nevertheless, our simulated results represent a lower bound on the average number of DSBs and their complexity. Our modeling approach, in fact, neglects the contribution of photons and Auger electrons emitted by ^177^Lu, the resonant formation of strand breaks by very low energetic electrons (<20 eV) (36–38), the induction of non-DSB oxidative clustered DNA lesions, and the consequence of sugar and base residue repair, which can increase the final strand break yield. In addition, cells exposed to ^177^Lu-DOTATATE are not synchronized in a specific cell-phase, as we assumed for the purpose of simplification, but are characterized by a distribution of radiosensitivity, associated with their cell phase, that should be accounted for when simulating different nuclei. Lastly, we did not include the possibility of DSB repair, given that repair mechanisms involved in TRT are not yet fully understood. Specifically, during TRT, since DNA damage induction persists over time, induction and repair occur simultaneously and hence repair mechanisms might differ significantly from EBRT. However, our approach might be justified by the very low DSB reduction pace (0.96% in 4 h) or, better, the substantial equilibrium between induction and DSB repair, indicated by the average (among the cell population) experimental decrease in the number of 53BP1 foci over 3 d (18). However, sublethal damage repair differs among the cell population depending on cell phase and dose rate variation too. Moreover, the DSBs are measured by means of 53BP1 foci—that is, repair foci—and hence might be slightly underestimated as well. Indeed, only breaks in which repair is induced are accounted for with this measurement, and the fluorescently labeled compound might not successfully bind to the 53BP1.

At present, only Eberlein et al. proved the existence of a correlation (with slope 0.0127 DSBs mGy^−1^ cell^−1^) between the absorbed dose to blood of patients undergoing ^177^Lu-DOTATATE treatment and the induction of DBSs, measured by the colocalized biomarkers γH2AX and 53BP1 (39). Remarkably, we found a similar number of DSBs per cell and per milligray (0.014 vs. 0.017 DSBs mGy^−1^ cell^−1^), which serves as further validation of our computational approach.

Further improvements in the computational chain pertain to the inclusion of base damage affecting the DSB complexity (40), different oxygen levels in the nuclear medium, different cellular shapes in a single-exposure scenario, intraorganelle variation in the internalized activity fraction, realistic cellular media, and a more representative distribution of the genetic material, according to the cell cycle and including realistic proportions of euchromatin and heterochromatin. Some of these improvements are currently being developed by the Geant4-DNA community and will be included in future simulations. More studies investigating the temporal variation in dose rates over time against biologic phenomena such as DNA repair capacity and cell cycle progression over the cell population would help to further improve biophysical modeling as well.

To develop a comprehensive model, not limited to a planar in vitro application but representative of an in vivo tumor scenario, a 3-dimensional aggregation of cells characterized by a variable SSTR_2_ expression should be modeled. For this purpose, the variability of SSTR_2_ expression among cellular population samples should be analyzed by means of fluorescence-activated cell sorting analysis or flow cytometry, so that the intensity of the receptor staining can be normalized and used to sample a heterogeneous receptor expression among the cell population. As such, various probability distributions of the SSTR_2_ expression can be generated to test the influence on the absorbed dose estimation. Moreover, the effect of ^177^Lu-DOTATATE on peritumoral vessels will influence tumor hemodynamics and, to a lesser extent, its cross-dose irradiation, which could be explicitly simulated by changing the proportion of activity bound to the vessels, according to tumor differentiation and aggressiveness. Somatostatin is known to cause vasoconstriction resulting in regional hypoxia or necrosis (41). The oxygen effect should be considered by modifying the chemistry processes (e.g., by adding the specific chemical processes that lead to the creation and the chemical reactions of radicals involving oxygen) or by simply correcting with the oxygen enhancement ratio for DSB induction. Finally, even though direct radiation effects will form the major contribution to cellular responses, bystander effects and abscopal effects should be studied in tissues with low-receptor expression. Indeed, bystander signaling may be present in receptor-negative cells within a matrix of receptor-positive cells but will be obscured by many other factors influencing cell survival. To model such effects, initial studies should be performed focused on selecting relevant radiation-perturbed molecular pathways or intracellular targets, which, when hit by radiation, initiate the emission of bystander signals (e.g., mitochondria, nuclear membrane, and ribosomes), in order to inform a more systemic description of the biologic response to radiation after TRT exposure.

## CONCLUSION

In this work, we developed a simulation framework to evaluate the number of DNA DSBs occurring during in vitro TRT, which, through further modifications and comparison with experimental data, can lead to a better understanding of the underlying biologic mechanisms of this therapy. Adopting this methodology, we found good agreement with experimental data and a clear correlation between the absorbed dose and the average number of DSBs per cell after ^177^Lu-DOTATATE exposure was established. Furthermore, this work highlights the importance of overcoming classic macrodosimetric approaches to be able to investigate and find correlations with the biologic response after TRT exposure, as is instrumental for personalized dosimetry.

## Notes

KEY POINTS **QUESTION:** Can a simulation framework be established to evaluate the number of DNA DSBs induced by in vitro TRT? **PERTINENT FINDINGS:** We found a linear correlation between the mean specific energy to the nucleus and the number of DSBs per cell: 0.014 DSBs per cell mGy^−1^ for internalization in G and 0.017 DSBs per cell mGy^−1^ for internalization in Cy. Furthermore, we found a spread in the induction of DSBs caused by the specific shape of the radioactive cell compartment and a significant difference in the DSB yields induced by bound and unbound activity fractions. **IMPLICATIONS FOR PATIENT CARE:** Ultimately, this simulation tool will elucidate the underlying biological mechanisms of TRT and interpret DNA-damage biomarkers. Knowledge of the physical, chemical, and biologic actions by TRT will lead the way to its optimization and a better response prediction in patients.
